# Inverse correlation between average monthly high temperatures and COVID-19-related death rates in different geographical areas

**DOI:** 10.1186/s12967-020-02418-5

**Published:** 2020-06-23

**Authors:** Francesca Benedetti, Maria Pachetti, Bruna Marini, Rudy Ippodrino, Robert C. Gallo, Massimo Ciccozzi, Davide Zella

**Affiliations:** 1 Institute of Human Virology, Department of Biochemistry and Molecular Biology, School of Medicine, University of Maryland, Baltimore, USA; 2 Elettra Sincrotrone Trieste - Area Science Park, Trieste, Italy; 3 Department of Physics, University of Trieste, Via Valerio 2, Trieste, Italy; 4 Ulisse BioMed - Area Science Park, Trieste, Italy; 5 Institute of Human Virology, Department of Medicine, School of Medicine, University of Maryland, Baltimore, USA; 6 Global Virus Network, Baltimore, USA; 7 Medical Statistic and Molecular Epidemiology Unit, University of Biomedical Campus, Rome, Italy

**Keywords:** SARS-CoV, SARS-CoV-2, COVID-19, Temperature, Latitude, Death rate

## Abstract

**Background:**

With the aim of providing a dynamic evaluation of the effects of basic environmental parameters on COVID-19-related death rate, we assessed the correlation between average monthly high temperatures and population density, with death/rate (monthly number of deaths/1 M people) for the months of March (start of the analysis and beginning of local epidemic in most of the Western World, except in Italy where it started in February) and April 2020 (continuation of the epidemic). Different geographical areas of the Northern Hemisphere in the United States and in Europe were selected in order to provide a wide range among the different parameters. The death rates were gathered from an available dataset. As a further control, we also included latitude, as a proxy for temperature.

**Methods:**

Utilizing a publicly available dataset, we retrieved data for the months of March and April 2020 for 25 areas in Europe and in the US. We computed the monthly number of deaths/1 M people of confirmed COVID-19 cases and calculated the average monthly high temperatures and population density for all these areas. We determined the correlation between number of deaths/1 M people and the average monthly high temperatures, the latitude and the population density.

**Results:**

We divided our analysis in two parts: analysis of the correlation among the different variables in the month of March and subsequent analysis in the month of April. The differences were then evaluated. In the month of March there was no statistical correlation between average monthly high temperatures of the considered geographical areas and number of deaths/1 M people. However, a statistically significant inverse correlation became significant in the month of April between average monthly high temperatures (p = 0.0043) and latitude (p = 0.0253) with number of deaths/1 M people. We also observed a statistically significant correlation between population density and number of deaths/1 M people both in the month of March (p = 0.0297) and in the month of April (p = 0.0116), when three areas extremely populated (NYC, Los Angeles and Washington DC) were included in the calculation. Once these three areas were removed, the correlation was not statistically significant (p = 0.1695 in the month of March, and p = 0.7076 in the month of April).

**Conclusions:**

The number of COVID-19-related deaths/1 M people was essentially the same during the month of March for all the geographical areas considered, indicating essentially that the infection was circulating quite uniformly except for Lombardy, Italy, where it started earlier. Lockdown measures were implemented between the end of March and beginning of April, except for Italy which started March 9th. We observed a strong, statistically significant inverse correlation between average monthly high temperatures with the number of deaths/1 M people. We confirmed the data by analyzing the correlation with the latitude, which can be considered a proxy for high temperature. Previous studies indicated a negative effect of high climate temperatures on Sars-COV-2 spreading. Our data indicate that social distancing measure are more successful in the presence of higher average monthly temperatures in reducing COVID-19-related death rate, and a high level of population density seems to negatively impact the effect of lockdown measures.

## Background

Recently a novel, human pathogen Severe Acute Respiratory Syndrome Coronavirus 2 (SARS-CoV-2) emerged in China and its worldwide spread created a global health emergency. The World Health Organization publicly declared the SARS-CoV-2 outbreak a pandemic on March 11th 2020, and so far the virus has caused more than 200,000 deaths worldwide, strongly impacting the global economy and human habits. Different environmental conditions can affect host–pathogen interactions. In particular most respiratory viruses are sensitive to local geographical conditions like local temperature and humidity [1]. This in turn generates seasonal waves of outbreaks, by impacting viral transmission and survival [2, 3].

Some studies indicate that warm climates influences SARS-CoV-2 geographical distribution by reducing its infectivity rates [4–9], and others include additional factors, like air pollution, as responsible for increased viral spread [10].

In this study we correlate the average monthly high temperatures and the population density of different geographical areas with number of deaths/1 M people. We focused our analysis on the number of deaths/1 M people instead of number of SARS-CoV-2 RNA-positive people to avoid potential bias due to the different number of total PCR tests in the different areas considered. The areas in the Northern hemisphere were selected to provide a wide range among the different parameters, and the death rate data were gathered from an available dataset. In order to provide a dynamic correlation, the average monthly high temperatures and deaths numbers data were compared for the months of March (start of the analysis and beginning of local epidemic in most of the Western World) with the month of April. Except in Italy, which started at the beginning of March, lockdown measures were implemented between the end of March and the beginning of April in the remaining areas. By reasoning that the latitude is a proxy for temperature, as a further control we then calculated the latitude of the same areas with the number of deaths/1 M. Our data may help to understand the impact of daily temperatures on COVID-19-related death rate and modeling the containment measures according to different geographical areas, and suggest that factors other than social practices contributed to the infection of SARS-CoV-2.

## Methods

Twenty-five areas located in Europe and in the United States have been selected as representative of the region/country where major outbreaks have been detected up to April 30th, 2020. We downloaded the number of deaths of confirmed COVID-19 cases of these areas [11] and we calculated the monthly number of deaths/1 M people. We then included in the dataset the average monthly high temperatures (°F) and latitude of these areas as climatic variables that can affect the spread and the lethality of COVID-19. The relationships among these variables have been analyzed with the R *tydiverse* package.

The data were obtained from the European Centre for Disease Prevention and Control (ECDC). We note that the criteria for defining death caused by or associated with COVID-19 are not homogenous internationally and therefore should be taken with caution [12, 13]. In addition, increased mortality indirectly related to COVID-19 may be indicative of sudden exhaustion of the hospital and health systems with a negative impact on the main mortality drivers (cancer, cardiovascular diseases and others). To overcome as much as possible these potential drawbacks, we selected geographical regions implementing homogenous parameters accounting for deaths related to COVID-19. More in detail, as it is standard in death reporting, Countries are asked to follow the “cause of death” classifications from the WHO’s *International Classification of Diseases* guidelines [14]. In addition, National guidelines are also implemented, with the aim to better define the cause of death as related to COVID-19. Although confirmed cases are reliant on a positive laboratory confirmation of the COVID-19 PCR test, a laboratory diagnosis may not be required for it to be listed as the cause of death. As a result, in some circumstances, depending on national guidelines, medical practitioners can record COVID-19 deaths if they think the signs and symptoms point towards this as the underlying cause.

## Results

We selected several geographical areas in the Northern hemisphere of the United States and Europe, based on providing a range among the different parameters considered. The States and regions considered are outlined in Table 1. Between parenthesis we indicated the locations selected to obtain the latitudes and temperatures used for our analysis. We compared the data related to the month of March 2020. Lockdown measures were implemented between the end of March and beginning of April, except in Italy which started earlier. The death rate data were gathered as described in the Methods section.

**Table 1:** Selected geographical areas

Location	Latitude	Average temperature March (°F)	Average temperature April (°F)
United Kingdom (London)	51.5	52.65	63.60
Belgium (Brussels)	50.84	51.16	63.80
Washington (Seattle)	47.6	50.65	60.17
Lombardy (Italy)	45.46	57.26	69.70
Massachusetts (Boston)	42.35	47.84	49.80
Michigan (Ann Harbor)	42.28	47.58	53.70
Illinois (Chicago)	41.88	47.71	56.27
Connecticut (Waterbury)	41.55	49.42	52.10
New Jersey (Newark)	40.73	54.52	57.70
New York (New York City)	40.71	53.94	56.60
Pennsylvania (Philadelphia)	39.95	56.48	59.27
Maryland (Baltimore)	39.29	59.65	61.73
Washington, D.C.	38.9	60.74	63.57
Sicily (Italy)	38.11	60.77	64.77
Malta (La Valletta)	35.93	62.77	68.53
New Mexico (Albuquerque)	35.08	62.58	71.23
California (Los Angeles)	34.05	67.74	73.40
Georgia (Atlanta)	33.74	69.00	70.77
Arizona (Phoenix)	33.44	73.55	86.45
South Carolina (Charleston)	32.77	71.48	75.60
California (San Diego)	32.71	63.90	67.40
Mississippi (Jackson)	32.3	75.13	74.37
Texas (Austin)	30.26	76.29	79.93
Louisiana (New Orleans)	29.95	78.94	80.60
Florida (Miami)	27.53	84.39	84.67

Between parenthesis are indicated the location considered for the latitude and to calculate the average monthly high temperatures

**Fig. 1:**
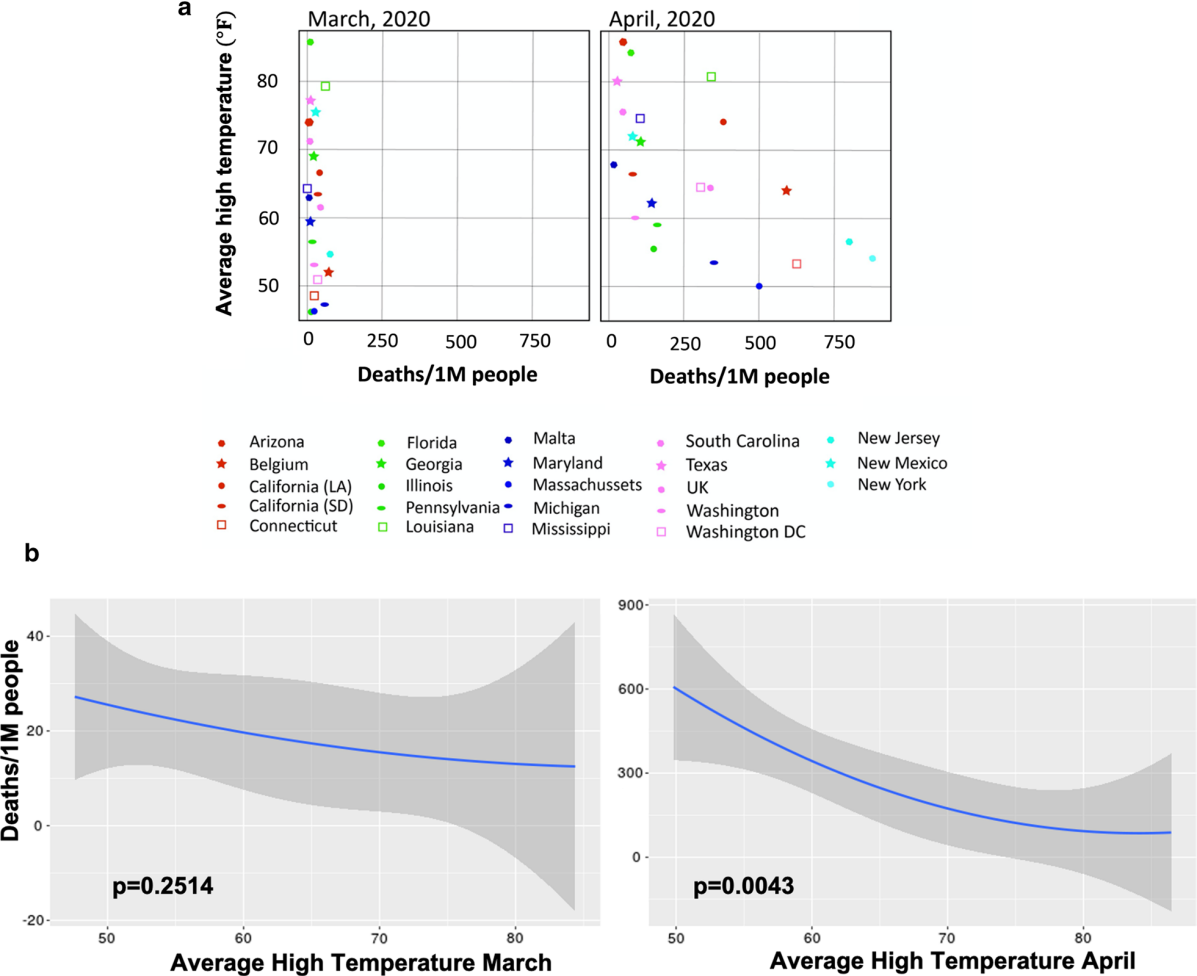
Correlation between average monthly high temperatures and number of deaths/1 M people. **a** The relationship between the number of deaths/1 M people and the average monthly high temperatures (°F) for the months of March (left) and April (right) 2020 is represented for 25 areas located in Europe and in the US using scatter plots graphs. **b** The not linear correlation between these two variables is calculated using the squared transformation of the average monthly high temperatures. The p-values for each month are indicated

First, we analyzed the correlation between number of deaths/1 M people and the average monthly high temperatures registered in each city considered (Fig. 1). The number of COVID-19-related deaths/1 M people was essentially the same during the month of March for all the geographical areas considered, indicating essentially that the infection was circulating quite uniformly (p = 0.2514). In April, we observed instead a significant increase in number of deaths/1 M people in those areas where the average monthly high temperatures registered were below 65 °F (Fig. 1a). We found a statistically significant correlation between the decreased number of deaths/1 M people with the increase of the average monthly high temperatures (p = 0.0043) (Fig. 1b). The same inverse correlation between average monthly high temperatures and COVID-19-related deaths was observed in two regions in Italy (Lombardy in the North and Sicily in the South, respectively) (Table 2). The difference was already noticeable in March, due to the early start of the epidemic, and become progressively accentuated in April (Table 2).

**Table 2:** Number of deaths per millions of people, average high temperatures for the months of March and April, and population density of two different Italian regions, Lombardy (North) and Sicily (South)

Location	Deaths/million of people in March	Deaths/million of people in April	Average high temperature in March (°F)	Average high temperature in April (°F)	Latitude (°)	Population density (sq ft)
Lombardy	500	700	57.26	69.70	45.46	420
Sicily	10	35	60.77	64.77	38.11	200

**Fig. 2:**
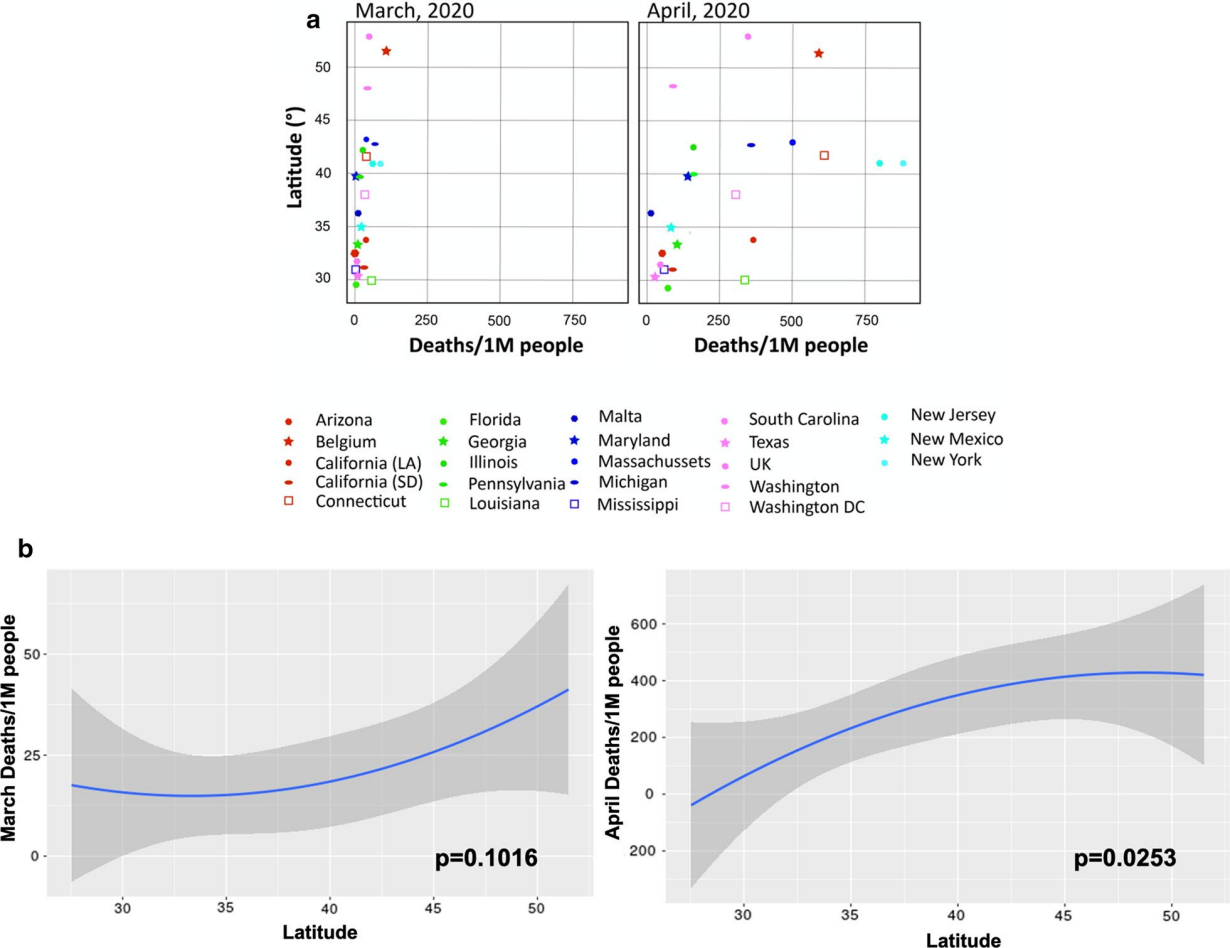
Correlation between latitude and number of deaths/1 M people. **a** The relationship between the number of deaths/1 M people and the latitude (used as a proxy of the average monthly high temperatures registered) for the months of March (left) and April (right) 2020 is represented for 25 areas located in Europe and in the US using scatter plots graphs. **b** The not linear correlation between these two variables is calculated using the squared transformation of the latitudes. The p-values for each month are indicated

We confirmed these data by analyzing the correlation between the number of deaths/1 M people and the latitude, considered as a proxy for the temperature, of each area (Fig. 2a, b). In March, we don’t see any statistically significant difference (p = 0.1016), while in April, the latitude of 40° is a clear separator: in areas located above 40° the number of deaths increase significantly compared to the number of deaths in areas located below 40° (p = 0.0253). The same inverse correlation between high latitude and COVID-19-related deaths was observed by comparing Lombardy to Sicily (Table 2).

**Fig. 3:**
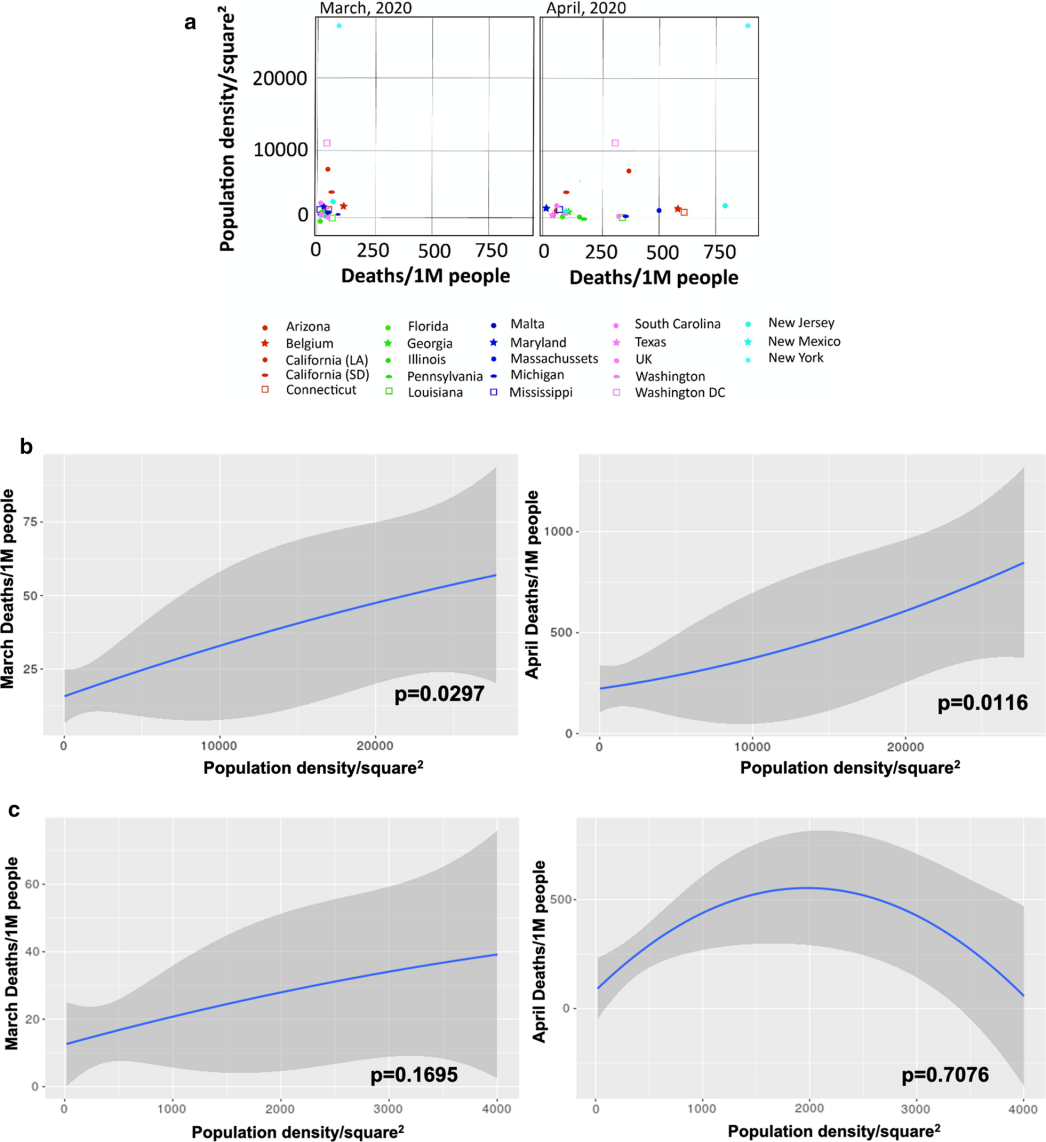
Correlation between population density and number of deaths/1 M people. **a** The relationship between the number of deaths/1 M people and the population density for the months of March (left) and April (right) 2020 is represented for 25 areas located in Europe and in the US using scatter plots graphs. **b** The not linear correlation between these two variables is calculated using the squared transformation of the population density including the three highly densely populated areas (New York City, Los Angeles and Washington, DC), and **c** excluding these areas. The p-values for each month are indicated

Finally, we observed a statistically significant correlation between population density and number of deaths/1 M people in March (Fig. 3a, b) (p = 0.0297) and April (p = 0.0116). However, this statistical significance is due to the presence of three areas with extremely high population density: New York City, Los Angeles (CA) and Washington DC (Fig. 3c). Once these three areas were removed, the correlation was not statistically significant (p = 0.1695 in the month of March, and p = 0.7076 in the month of April).

In conclusion, our data indicate that there is a strong correlation between the number of deaths/1 M people and climate. It is likely that social distancing measure are more successful in the presence of average monthly high temperatures in reducing COVID-19-related death rate, and high level of population density seems to reduce the effect of lockdown measures.

## Discussion

We correlate the average monthly high temperatures and the population density of different geographical areas with number of deaths/1 M people. The data were analyzed for the month of March and April.

We did not find any correlation between temperatures and number of deaths in the month of March. On the contrary, there was a strong statistical correlation between the temperature values and number of deaths in the month of April. These data were further confirmed when we used latitude as a proxy of the temperature. It is noteworthy to remember that in all the areas considered, lockdown measures were implemented between the end of March and beginning of April (except in Lombardy and Sicily, Italy, which started earlier), and all had substantial forms of social distancing by April. Our results indicate that the areas considered started from a very similar number of deaths in the month of March, but the implementation of social distancing seems to be more successful in those areas with higher average monthly temperatures during the month of April.

We could not find a correlation between population density and number of deaths once the especially densely populated areas were removed. These data seem to indicate a possible threshold of population density as a factor contributing to virus spread.

## Conclusion

Our data indicate that social distancing measures are more successful in the presence of higher daily temperature in reducing COVID-19-related death rates. On the contrary, high level of population density seems to reduce the effect of lockdown measures. Though more studies are necessary to confirm these data, we believe they help understand the impact of temperature on COVID-19-related death rate and modeling the containment measures according to different geographical areas.

## Data Availability

All data utilized, generated or analyzed during these studies are included in this published article.

## Abbreviations

SARS-CoV: Severe acute respiratory syndrome coronavirus
SARS-CoV-2: Severe acute respiratory syndrome coronavirus 2 (COVID-19)
MERS-CoV: Middle east respiratory syndrome coronavirus
WHO: World Health Organization
COVID-19: Coronavirus Disease-19
